# REST is a major negative regulator of endocrine differentiation during pancreas organogenesis

**DOI:** 10.1101/gad.348501.121

**Published:** 2021-09-01

**Authors:** Meritxell Rovira, Goutham Atla, Miguel Angel Maestro, Vane Grau, Javier García-Hurtado, Maria Maqueda, Jose Luis Mosquera, Yasuhiro Yamada, Julie Kerr-Conte, Francois Pattou, Jorge Ferrer

**Affiliations:** 1 Department of Physiological Science, School of Medicine, Universitat de Barcelona (UB), L'Hospitalet de Llobregat, Barcelona 08907, Spain;; 2 Pancreas Regeneration: Pancreatic Progenitors and Their Niche Group, Regenerative Medicine Program, Institut d'Investigació Biomèdica de Bellvitge (IDIBELL), L'Hospitalet de Llobregat, Barcelona 08908, Spain;; 3 Program for Advancing the Clinical Translation of Regenerative Medicine of Catalonia (P-CMR[C]), L'Hospitalet de Llobregat, Barcelona 08908, Spain;; 4 Center for Networked Biomedical Research on Bioengineering, Biomaterials, and Nanomedicine (CIBER-BBN), Madrid 28029, Spain;; 5 Regulatory Genomics and Diabetes, Centre for Genomic Regulation, Barcelona Institute of Science and Technology, Barcelona 08003, Spain;; 6 Centro de Investigación Biomédica en Red Diabetes y Enfermedades Metabólicas Asociadas (CIBERDEM), Madrid 28029, Spain;; 7 Bioinformatics Unit, Bellvitge Biomedical Research Institute, IDIBELL, L'Hospitalet del Llobregat, Barcelona 08908, Spain;; 8 Division of Stem Cell Pathology, Center for Experimental Medicine and Systems Biology, Institute of Medical Science, University of Tokyo, Tokyo 108-8639, Japan;; 9 Institute Pasteur Lille, University of Lille, Institut National de la Santé et de la Recherche Médicale (INSERM), Centre Hospitalier Universitaire de Lille (CHU Lille), U1190, European Genomic Institute for Diabetes (EGID), Lille F-59000, France;; 10 Department of Metabolism, Digestion, and Reproduction, Section of Genetics and Genomics, Imperial College London, London W12 0NN, United Kingdom

**Keywords:** REST, β cells, bipotent progenitors, endocrine differentiation, pancreas, pancreas development, transcriptional repressors

## Abstract

Multiple transcription factors have been shown to promote pancreatic β-cell differentiation, yet much less is known about negative regulators. Earlier epigenomic studies suggested that the transcriptional repressor REST could be a suppressor of endocrinogenesis in the embryonic pancreas. However, pancreatic *Rest* knockout mice failed to show abnormal numbers of endocrine cells, suggesting that REST is not a major regulator of endocrine differentiation. Using a different conditional allele that enables profound REST inactivation, we observed a marked increase in pancreatic endocrine cell formation. REST inhibition also promoted endocrinogenesis in zebrafish and mouse early postnatal ducts and induced β-cell-specific genes in human adult duct-derived organoids. We also defined genomic sites that are bound and repressed by REST in the embryonic pancreas. Our findings show that REST-dependent inhibition ensures a balanced production of endocrine cells from embryonic pancreatic progenitors.

## Abstract (precis)

In this study, Rovira et al. report that inactivation of the transcriptional repressor REST causes a drastic increase in pancreatic endocrine progenitors and endocrine cells, and establish that REST is a major negative regulator of embryonic pancreas endocrine differentiation in mice and zebrafish. Their findings show that REST-dependent inhibition ensures a balanced production of endocrine cells from embryonic pancreatic progenitors.

Progress in our understanding of the transcriptional mechanisms underlying pancreatic β-cell differentiation has been crucial for recent advances in the development of regenerative therapy strategies for type 1 diabetes mellitus, including efforts to generate functional β cells from stem cells, organoids, or in vivo reprograming (Rezania et al. 2014; Huch and Koo 2015; Zhou and Melton 2018). Pancreatic islet cell transcription is also central to the mechanisms that underlie various forms of diabetes (Servitja and Ferrer 2004; Guo et al. 2013; Miguel-Escalada et al. 2019).

Cellular programming and differentiation result from an interplay of positive and negative transcriptional regulatory mechanisms (Crews and Pearson 2009; Graf and Enver 2009). Several DNA binding transcription factors are known to promote endocrine differentiation during pancreas development (Sussel et al. 1998; Gradwohl et al. 2000; Osipovich et al. 2014; De Vas et al. 2015). A more limited number of transcriptional regulators, such as HES1 and TEAD-YAP, have been shown to exert negative endocrine regulation (Jensen et al. 2000; Cebola et al. 2015; Mamidi et al. 2018). Some lines of evidence have also suggested that the RE-1 silencing transcription factor (REST; also known as NRSF, for neural-restrictive silencing factor) could be a negative regulator of endocrine differentiation during pancreas development (van Arensbergen et al. 2010).

REST is best known for its role as a suppressor of neuronal genes in nonneuronal cell types (Schoenherr and Anderson 1995). It binds a 21-bp DNA recognition sequence and has two repressor domains that recruit corepressor complexes. Consistent with its function to inhibit neuronal genes, REST is largely expressed in nonneuronal cell types. However, REST is not expressed in endocrine cell lines, and several genes that are repressed by REST are active in islet cells (Atouf et al. 1997; Martin et al. 2008, 2012). Furthermore, genome-wide studies in embryonic stem cells and nonneuronal cell types have shown direct binding of REST near β-cell-enriched genes (Johnson et al. 2008; van Arensbergen et al. 2010; Mukherjee et al. 2016). REST binding sites in embryonic stem cells overlap with genomic regions that carry Polycomb-repressed chromatin in FACS-purified multipotent progenitors of the early embryonic pancreas (van Arensbergen et al. 2010). Many of these Polycomb-repressed regions are β-cell regulatory genes that are subsequently derepressed during pancreatic endocrine differentiation, in parallel with the concomitant loss of REST expression (van Arensbergen et al. 2010). These correlations suggested that REST could be an important negative regulator of the endocrine differentiation program of the developing pancreas. A recent report exploited this notion by inhibiting REST to enhance PDX1-mediated activation of endocrine genes in adult pancreatic exocrine cells (Elhanani et al. 2020).

Genetic loss-of-function studies, however, failed to support a significant role of REST in pancreatic endocrine differentiation. Cre/LoxP-based excision of *Rest* in pancreatic progenitors led to changes in the expression of some endocrine genes but did not affect the number of endocrine cells, suggesting it was not an essential modulator of endocrine differentiation (Martin et al. 2015). Another pancreas deletion study reported that REST tempers pancreatic tissue damage and prevents acinoductal metaplasia, but the study did not explicitly assess endocrine differentiation (Bray et al. 2020). These studies, however, used an allele that removes *Rest* exon 2 (Gao et al. 2011). Recent work using a gene trap that disrupts transcription from all *Rest* promoters revealed dramatic effects on embryonic neurogenesis that were not observed when targeting *Rest* exon 2 (Nechiporuk et al. 2016). The same study showed that excision of *Rest* exon 2 does not prevent translation of a C-terminal REST peptide that is able to bind DNA, recruit corepressors, and repress target genes (Nechiporuk et al. 2016). Existing data, therefore, warrant a need to explore the true impact of REST in pancreatic endocrine differentiation using alternative genetic tools.

We have now inactivated *Rest* in the embryonic pancreas using a conditional allele that led to a marked increase in endocrine differentiation, proliferation, and cell mass. Inactivation of *Rest* in adult mature duct cells, however, failed to elicit this effect. We used chemical inhibitors to show that REST function is conserved in zebrafish and represses endocrine genes in human pancreas organoids. Finally, we defined key properties of the REST-dependent program during pancreas organogenesis. Our results, therefore, show an essential role of REST as a major negative regulator of pancreatic endocrine differentiation.

## Results

### REST expression in pancreas is largely restricted to progenitors and duct cells

The expression of REST in pancreatic cell types has been difficult to resolve unequivocally owing to low expression levels and lack of robust antibodies (van Arensbergen et al. 2010; Martin et al. 2015). We found nuclear REST immunoreactivity in most nonendocrine epithelial and mesenchymal cells of the mouse E12.5 pancreas, whereas from E14.5 onward it was largely restricted to duct-like clusters, and absent from acinar and endocrine cell clusters (Supplemental Fig. S1A). Purified duct cells from adult and E18.5 *Sox9*-eGFP transgenic mice (Gong et al. 2003) confirmed *Rest* mRNA expression in duct cells (Supplemental Fig. S1B,C). Finally, single-cell RNA-seq data (Tabula Muris et al. 2018) showed *Rest* mRNA in adult duct and nonepithelial cells but not in acinar or endocrine cells (Supplemental Fig. S1D). These results reinforce the notion that REST is expressed in embryonic bipotent progenitors and adult pancreatic ductal cells but is not detected in endocrine cells, consistent with a potential function of REST as a negative regulator of pancreatic endocrine differentiation.

### REST inactivation in pancreatic progenitors induces *Neurog3*

Previous genetic studies concluded that genetic ablation of *Rest* in pancreatic multipotent progenitors has no impact on the formation of NEUROG3+ endocrine precursors or hormone-producing cells (Martin et al. 2015), although this was examined in a mouse model that creates a deletion of *Rest* exon 2, which produces a functional isoform that can still bind DNA and recruit corepressors (Nechiporuk et al. 2016). We thus used an allele that enables conditional excision of exon 4, which encodes >75% of REST protein residues (Yamada et al. 2010). Breeding this line with a *Pdx1*-Cre transgene (Gu et al. 2002) enabled the excision of *Rest* and a severe depletion of REST protein in most embryonic pancreatic epithelial cells (hereafter referred to as *Rest*^pKO^ mice) (Supplemental Fig. S2A–C).

**Figure 1.**
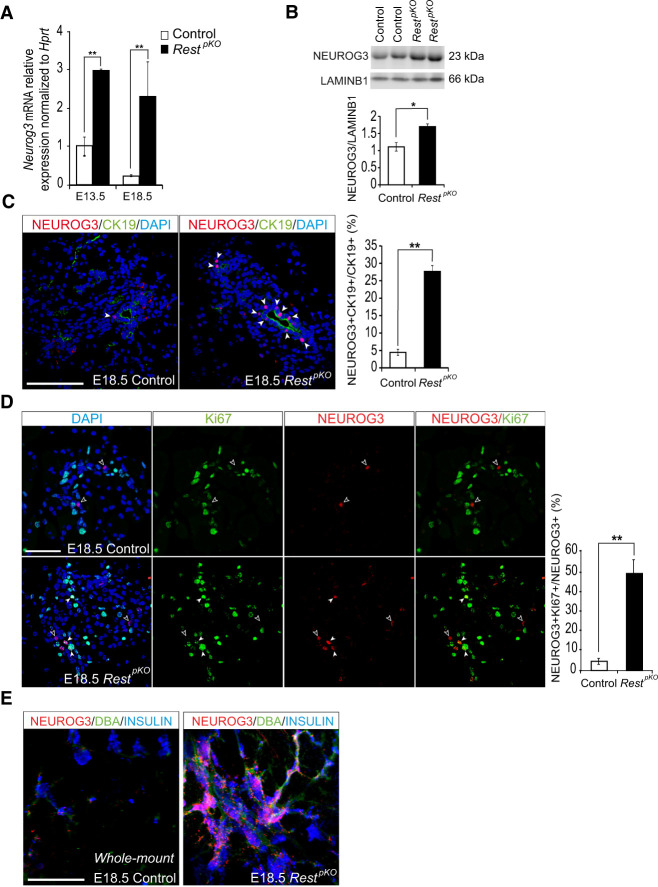
*Rest* inactivation in pancreatic progenitors induces NEUROG3. (*A*) *Neurog3* mRNA increases in E13 and E18 *Rest*^pKO^ pancreas. Normalization by *Hprt* mRNA; *n* = 3–4 mice per group. (*B*) Western blot and quantifications of NEUROG3 in nuclear extracts from E13.5 control and *Rest*^*pKO*^ pancreas. LamininB1 was used as loading control. *n* = 2 samples per group with a pull of three E13.5 pancreas per sample. (*C*) Immunofluorescence for NEUROG3 (red), cytokeratin 19 (CK19; green), and DAPI (blue) in E18.5 control and *Rest*^pKO^ pancreas. Arrowheads indicate NEUROG3+ cells. Bars show NEUROG3+ CK19+ cells in E18.5 pancreas. Scale bar, 100 µm. (*D*) Immunofluorescence for NEUROG3 (red), Ki67 (green), and DAPI (gray) in E18.5 pancreas. Empty arrowheads indicate NEUROG3+Ki67− cells, and white arrowheads indicate NEUROG3+Ki67+ cells. *n* = 4–6 mice per group. Scale bar, 50 µm. (*E*) Representative whole mounts for NEUROG3+ (red), DBA (green), and insulin (blue) of the tail of E18.5 control and *Rest*^*pKO*^ pancreas. Scale bars, 100 µm. Error bars are SEM. (*) *P* ≤ 0.05, (**) *P* ≤ 0.01.

Previous work showed that REST binds to pancreatic endocrine regulatory genes in mouse ES cells and that during embryonic pancreas differentiation, REST target genes loose Polycomb-repressed chromatin and undergo transcriptional activation (van Arensbergen et al. 2010). To directly test whether this means that REST truly acts as a repressor of endocrine differentiation during pancreas development, we examined expression of the endocrine lineage-determinant NEUROG3 in *Rest*^pKO^ embryos. This showed a 3.0-fold ± 0.03-fold increase of *Neurog3* mRNA in pancreas from *Rest*^pKO^ versus control E13.5 embryos (SEM, Student's *t*-test *P* < 0.01) and 1.7-fold increased NEUROG3 protein (*P* < 0.05) (Fig. 1A,B). At E18.5, a time point in which the wave of NEUROG3+ cells have normally begun to wane, *Neurog3* mRNA was 10.7-fold ± 1.2-fold higher in *Rest*^pKO^ embryos (*P* < 0.01) (Fig. 1A). Furthermore, E18.5 mutant pancreas showed an approximately sixfold increase in NEUROG3+ cells normalized by the total number of CK19^+^ cells (*P* < 0.01) (Fig. 1C). Several NEUROG3+ cells in mutant pancreas appeared to line the epithelium of large ducts (Fig. 1C). Therefore, REST inactivation in pancreatic progenitors led to an increased yield of NEUROG3+ cells throughout embryogenesis.

During normal pancreas development, the activation of *Neurog3* is followed by cell cycle exit of most progenitor cells (Miyatsuka et al. 2011; Krentz et al. 2017). This change in cell cycle activity has been shown to occur in discrete, strongly expressing NEUROG3+ cells, in contrast to remaining bipotent progenitor cells, which also express Neurog3 mRNA but very low, often undetectable NEUROG3 protein (Villasenor et al. 2008). We therefore investigated if REST inactivation affected the proliferation of discrete NEUROG3-expressing cells. Immunofluorescence analysis of E18.5 *Rest*^*pKO*^ pancreas showed that 48.3% ± 6.8% of NEUROG3+ cells coexpressed Ki67 versus 4.6% ± 1.5% in control embryos (*P* < 0.01) (Fig. 1D). This suggests that REST acts as a negative regulator of cell cycle exit in NEUROG3+ cells, which could contribute in part to the increased number of NEUROG3+ cells in *Rest*^*pKO*^ embryonic pancreas.

During embryogenesis, NEUROG3+ cells arise from pancreatic progenitors that form a tubular plexus that progressively evolves into a ductal tree (Gu et al. 2002; Solar et al. 2009; Bankaitis et al. 2015). We tested whether *Rest* deficiency could not only increase the yield of NEUROG3+ cells during embryonic development but also cause persistent *Neurog3* activation in the duct epithelium throughout postnatal life. We therefore examined postnatal (2-wk-old and 12-wk-old) *Rest*^pKO^ mice yet found no NEUROG3+ cells (Supplemental Fig. S3). This suggests that additional REST-independent mechanisms are required for persistent *Neurog3* activation during postnatal life.

Our studies, therefore, show that REST tempers the yield of NEUROG3+ cells in the embryonic pancreas, in part by suppressing NEUROG3+ cell proliferation. The lack of NEUROG3+ cells in the adult *Rest*^*pKO*^ pancreas suggests that REST function is not required to prevent the continuous formation of endocrine progenitor cells from duct cells during postnatal life.

### Increased β-cell mass in mice lacking REST in pancreatic progenitors

Given that the inactivation of REST in pancreatic progenitors led to the expansion of endocrine-committed NEUROG3+ progenitors, we investigated how this influenced the formation of endocrine cells. Whole-mount stainings for NEUROG3, CK19, and insulin in E18.5 pancreas not only confirmed increased NEUROG3+ cells but also showed a marked increase of the number of insulin-expressing cells (Fig. 1E).

**Figure 2.**
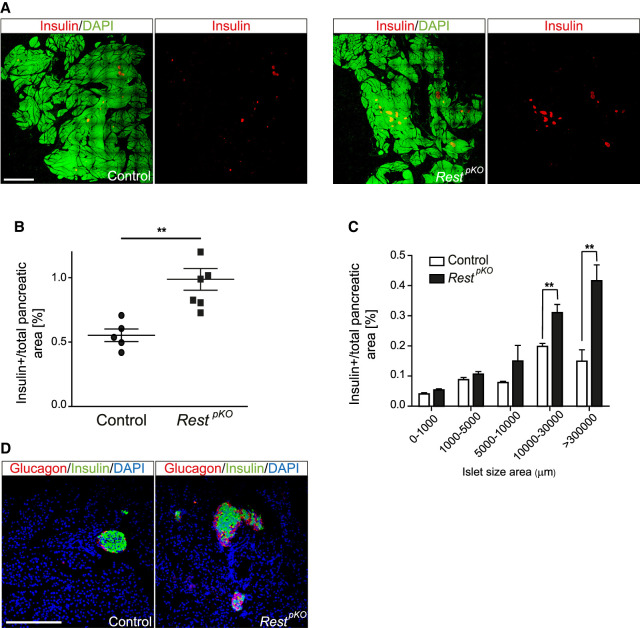
Increased β-cell mass in *Rest*^pKO^ mice. (*A*) Representative images of 10 × 10 frame reconstructions used for β-cell morphometry of insulin (red) and DAPI (green) stainings in pancreas from 12-wk-old control and *Rest*^pKO^ mice. Scale bar, 2 mm. (*B*) Morphometry of β-cell mass estimated from insulin surface area/total DAPI surface area (percentage). *Rest*^pKO^ mice have an approximately twofold increase in β-cell mass. *n* = 12 sections from five to six mice in each group. (*C*) Islet size in control and *Rest*^pKO^ adult pancreas. (*D*) Representative immunofluorescence for glucagon (red), insulin (green), and DAPI (blue) in whole-pancreas from 12-wk-old control and *Rest*^pKO^ pancreas. Scale bar, 200 µm. Error bars indicate SEM. (**) *P* ≤ 0.01.

Because the increase in NEUROG3+ cells observed in *Rest*^pKO^ pancreas was transient, we investigated whether increased β-cell mass was maintained in the adult pancreas. Young (12- to 16-wk-old) male *Rest*^*pKO*^ mice had normal weight (24.39 g ± 0.5 vs. 24.36 g ± 0.6 control mice) and fasting glycemia (49.71 mg/dL ± 1.83 vs. 54.26 mg/dL ± 3.44 control mice). Morphometric analysis, however, showed an approximately twofold increase of β-cell mass in 12-wk-old *Rest*^pKO^ versus control mice, which was primarily owing to an increase of large islets (Student's *t*-test, *P* < 0.01), as well as an apparent increase in glucagon-expressing cells (Fig. 2A–D). These findings, therefore, showed that inactivation of *Rest* in pancreatic progenitors results in a transient expansion of NEUROG3+ cells and a sustained increase of pancreatic endocrine cell mass.

### REST is a direct regulator of pancreatic endocrine differentiation

**Figure 3.**
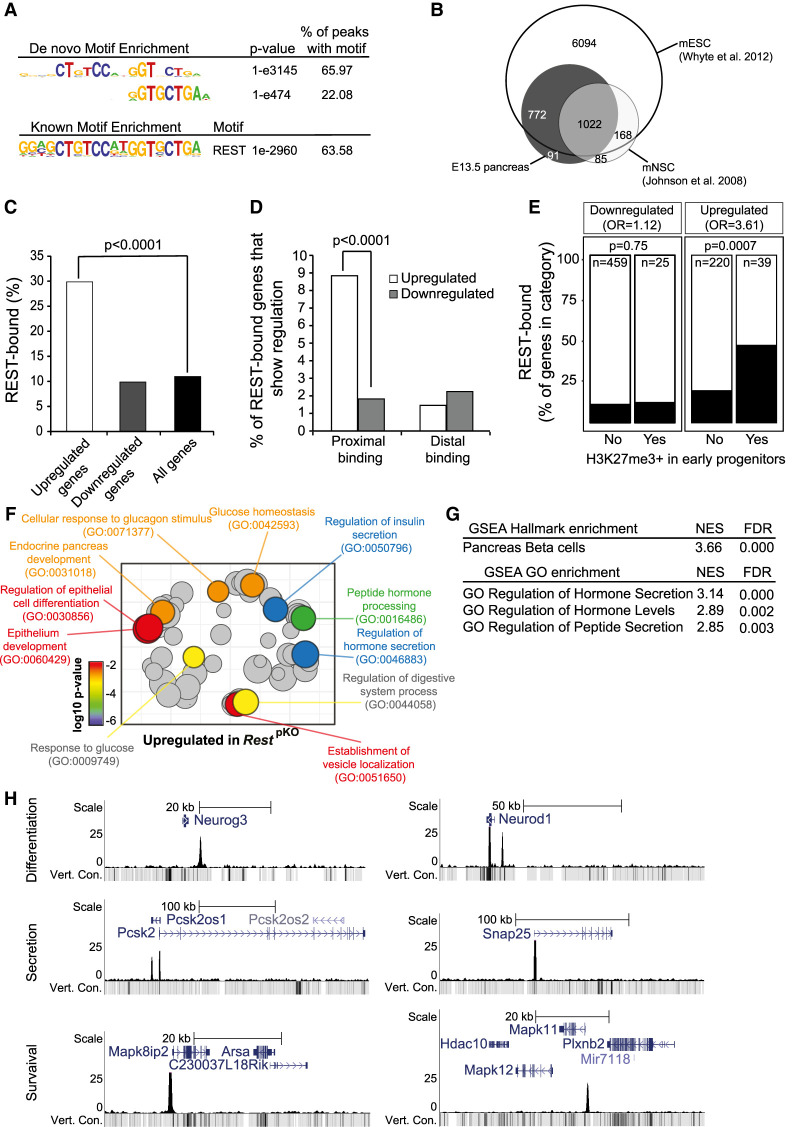
Functional direct REST targets in the embryonic pancreas. (*A*) Top de novo and known motif enrichments in REST-bound regions. (*B*) REST-bound regions in E13.5 pancreas, mESCs, and mNSCs. (*C*) Percentage of up-regulated and down-regulated genes in *Rest*^pKO^ mice, or all genes, that were bound by REST. Results indicate that REST predominantly acts as a repressor. *P*-values are Fisher exact test. (*D*) REST binds preferentially to promoter-proximal (0- to 5-kb) regions of genes that were up-regulated in *Rest*^pKO^ mice. (*E*) Percentage of differentially expressed genes that were bound by REST, broken down by H3K27me3 enrichment in purified pancreatic progenitors in (van Arensbergen et al. 2010). REST binding was enriched in genes that were up-regulated in *Rest*^pKO^ and showed H3K27me3 in progenitors. *P*-values from Fisher exact test. (*F*) Up-regulated genes were functionally annotated using Gorilla (Eden et al. 2009), and REVIGO (Supek et al. 2011) was used to visualize annotation clusters. The most significant terms are highlighted according to a *P*-value color scale. (*G*) Significant GSEA terms for up-regulated genes. (*H*) REST binding associated to pancreatic endocrine development (*Neurog3* and *NeuroD1*), insulin secretion (*Snap25* and *Pcsk2*), and β-cell survival (*Mapk11* and *Mapk8ip2*) genes in E13.5 pancreas. *Y*-axes are −log_10_ *P*-values. The vert. cons. track depicts vertebrate conservation.

Several genome-wide studies show that REST binds and regulates different genes in different cell types (Bruce et al. 2009; Hwang and Zukin 2018), although REST binding sites have not yet been mapped in pancreas. To study how REST controls pancreatic endocrine differentiation, we identified REST genomic binding sites and REST-dependent transcriptional changes in embryonic pancreatic progenitors. We performed ChIP-seq analysis using a monoclonal antibody (12C11) directed to the REST C-terminal region (Chen et al. 1998), and used chromatin from E13.5 wild-type pancreas, a stage at which REST expression is largely confined to progenitor cells and interspersed mesenchymal cells (Supplemental Fig. S1A). We detected 1968 REST-bound regions (Supplemental Table S1). These were highly enriched in canonical REST recognition motifs, confirming the specificity of REST binding (Supplemental Table S2; Fig. 3A).

The binding properties of repressors are poorly understood. Integration with ATAC-seq profiles from E13.5 pancreas revealed that REST-bound regions were accessible to transposase cleavage yet had an accessibility footprint that was narrower than recognition sites of activating pancreatic transcription factors, plausibly because active regulatory elements are occupied by multiple DNA binding factors (Supplemental Fig. S4).

DNA binding transcription factors that are expressed in multiple cell types often bind to different genomic regions across cell types (e.g., see Servitja et al. 2009), and this has also been observed for REST (Bruce et al. 2009; Hwang and Zukin 2018). We found that 1806 (∼94%) of REST-bound sites in embryonic pancreas were shared with embryonic stem cells but only 1030 (∼53%) with neuronal stem cells (Johnson et al. 2008; Whyte et al. 2012), whereas 91 (∼4.7%) were exclusively detected in mouse embryonic pancreas (Fig. 3B).

These findings, therefore, defined direct REST-bound regions in early embryonic pancreas. They confirmed that numerous bound regions vary across cell types, although the vast majority are shared with embryonic stem cells (Supplemental Table S3).

To further assess REST function in pancreatic progenitors, we compared RNA-seq profiles of E18.5 *Rest*^pKO^ versus control pancreas, a stage at which many progenitors have already been allocated to distinct cellular lineages. We identified 484 down-regulated and 259 up-regulated genes in *Rest*^pKO^ E18.5 embryonic pancreas (adjusted *P* < 0.05) (Supplemental Table S4). We found that 29.8% of up-regulated genes were bound by REST (Fisher *P* < 10^−4^, relative to 15,548 expressed genes), whereas only 9.8% of down-regulated genes were bound, a similar frequency as all expressed genes (11.2%; Fisher *P* = 0.81) (Fig. 3C; Supplemental Table S5). This was consistent with the notion that the *Rest*^pKO^ phenotype reflects a transcriptional repressor function of REST in the developing pancreas.

REST binds to distal and proximal genomic sites, both of which are likely to harbor functional relevance. However, up-regulated genes in *Rest*^pKO^ were most strongly enriched among genes with promoter-proximal REST binding (Fisher *P* < 10^4^) (Fig. 3D), which was also observed in early experiments that examined dominant negative inhibition of REST in mouse ESCs (Johnson et al. 2008) Furthermore, REST-bound up-regulated genes were enriched in Polycomb-repressed chromatin in pancreatic progenitors (odds ratio = 3.61, Fisher *P* = 7 × 10^−4^ for up-regulated REST-bound genes; odds ratio = 1.12, *P* = 0.75 for down-regulated REST-bound genes; both calculated relative to H3K27me3-enriched genes in PDX1+ E10.5 pancreatic progenitors as defined by van Arensbergen et al. 2010) (Fig. 3E).

Consistent with the increase in endocrine cells in *Rest*^pKO^ pancreas, genes that were up-regulated, as well as pancreas REST-bound genes at large, showed a strong enrichment in pancreatic endocrine annotations, including insulin secretion and processing, glucose homeostasis, and endocrine pancreas development (Fig. 3F,G; Supplemental Tables S6, S7). Closer inspection of individual loci disclosed the location of REST-bound regions at important regulators of pancreatic differentiation (*Neurog3*, *Neurod1*, *Insm1*, *Hnf4a*, *Onecut1*, *Pax4*, *Glis3*, and *Hnf1a*), insulin biosynthesis or exocytosis (*Pcsk1*, *Pcsk2*, *Scg3*, *Snap25*, and *Syt7*), as well as endocrine cell growth (*Bid*, *Mapk8IP1*, *Mapk10*, and *Mapk11*) (Fig. 3H; Supplemental Table S3). Regions bound by REST in embryonic pancreas but not in neural or even embryonic stem cells included genes previously associated with pancreatic differentiation and function such as *Isl1* (Ahlgren et al. 1997), *Prox1* (Paul et al. 2016), or *Cdh13* (Supplemental Fig. S5; Tyrberg et al. 2011). Given the increased proliferation of NEUROG3+ cells, it was also interesting to note up-regulation of REST-bound positive cell cycle regulators in *Rest*^pKO^, including *Cdk5r2*, *Cdk2ap1*, *Ccnd1*, *Mapk3*, and *Ret* (Supplemental Fig. S5; Supplemental Table S5). These studies, therefore, identified direct target genes through which REST controls pancreatic endocrine differentiation programs.

### Postnatal inactivation of REST

**Figure 4.**
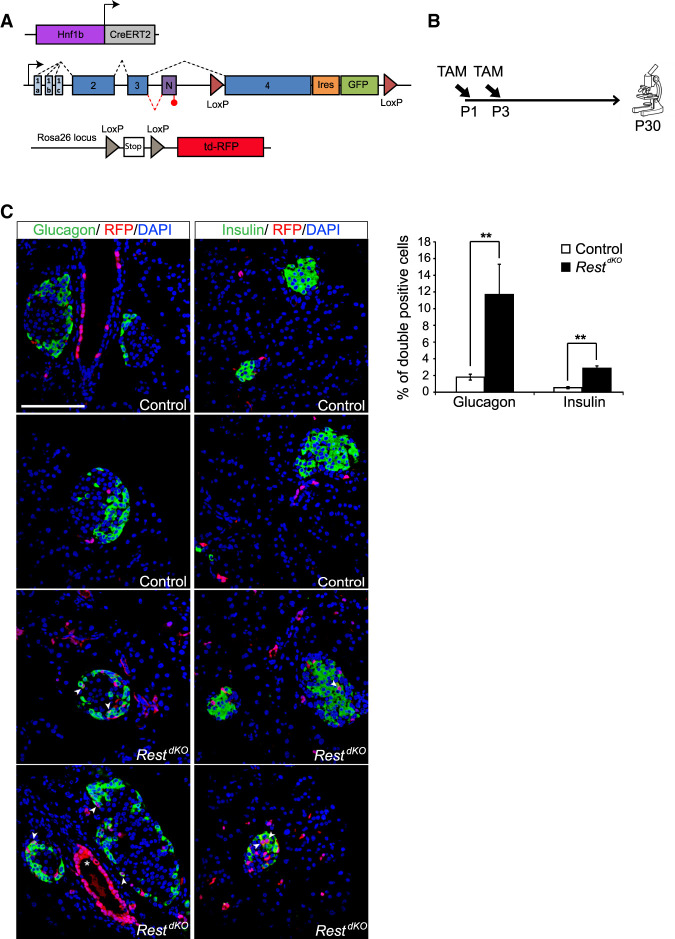
Pancreas-specific inactivation of Rest in neonatal ducts. (*A*) Schematic of genetic models used to inactivate *Rest* and activate RFP expression in duct cells and progeny. *Hnf1b*-CreERT2 is a BAC transgenic that specifically marks duct cells (Solar et al. 2009), as well as non-*Rest*-expressing ∂ cells in reporters that are excised with high efficiency (Rovira et al. 2021), but not other endocrine or acinar cells. (*B*) Schematic of the lineage tracing experiment. Tamoxifen was given to mothers at day 1 (P1) and day 3 (P3) after delivery, and mice were analyzed at P30. *Hnf1b*-CreERT2;Rosa26^RFP^ control mice were also treated. (*C*) Representative images of double-positive RFP (red) and insulin (green) cells and of double-positive RFP (red) and glucagon (green) cells in *Rest*^dKO^ and control mice. The graph shows RFP-expressing glucagon and insulin cells in *Rest*^dKO^ versus control mice. *n* = 5–6 mice per each group. Arrowheads indicate double-positive cells, and an asterisk marks examples of cells in a duct, which were very efficiently labelled. Scale bar, 100 µm. Error bars are SEM. Student's *t*-test; (**) *P* < 0.01.

Embryonic duct-like bipotent progenitors express many duct cell markers, as well as progressively lose their progenitor capacity as they mature to differentiated duct cells (Solar et al. 2009; Kopinke et al. 2011; Kopp et al. 2011; Bankaitis et al. 2015) REST expression, however, is maintained as embryonic progenitors transition to adult differentiated ductal cells (Supplemental Fig. S1). We therefore asked whether REST inactivation immediately after birth could increase the capacity for de novo generation of endocrine cells. To this end, we used the *Hnf1b*-CreERT2transgenic line (Solar et al. 2009) to excise the *Rest* LoxP allele in HNF1B+ cells (most of which are duct cells), and also used a Rosa26-LSL-RFP reporter (Luche et al. 2007) to trace the progeny of cells that have undergone *Rest* excision (Fig. 4A). We treated dams of triple transgenic newborns (hereafter called *Rest*^dKO^ Rosa26^RFP^) or *Hnf1b*-CreERT2;Rosa26^RFP^controls with tamoxifen at postnatal days 1 and 3 and then analyzed mice after weaning (Fig. 4B). This showed that the number of insulin+/RFP+ and glucagon+/RFP+ cells was increased 5.3-fold ± 0.5-fold and 6.4-fold ± 1.6-fold (Student's *t*-test *P* < 0.01), respectively, in *Rest*^dKO^ mice relative to *Hnf1b*-CreERT2;Rosa26^RFP^ control mice (Fig. 4C). These results indicate that although the inactivation of REST in embryonic pancreatic progenitors did not result in persistent activation of endocrine progenitor markers throughout adult life, induced inactivation of REST in neonatal pancreas did transiently increase endocrine cell formation.

In contrast, REST inactivation in pancreatic duct cells from 12 wk olds using the same lineage tracing model did not lead to significantly increased number of insulin+/RFP+ cells 4 wk after induction (2.54% ± 0.33% in *Rest*^dKO^ Rosa26^RFP^ vs. 1.96% ± 0.49% in control mice; Student's *t*-test, *P* = 0.177) (Supplemental Fig. S6). Thus, although REST retains an essential function to suppress endocrine cell formation in early postnatal periods, this role subsides in adult mice, consistent with a more limited differentiation potency of mature duct cells.

### Chemical inhibition of REST in zebrafish

Encouraged by the observation that inactivation of REST in early postnatal duct cells increased the de novo generation of endocrine cells, we explored whether similar effects could be extended to other model systems using chemical inhibition of REST. We used X5050, recently identified in a high-throughput screen to inhibit REST by protein destabilization (Charbord et al. 2013).

**Figure 5.**
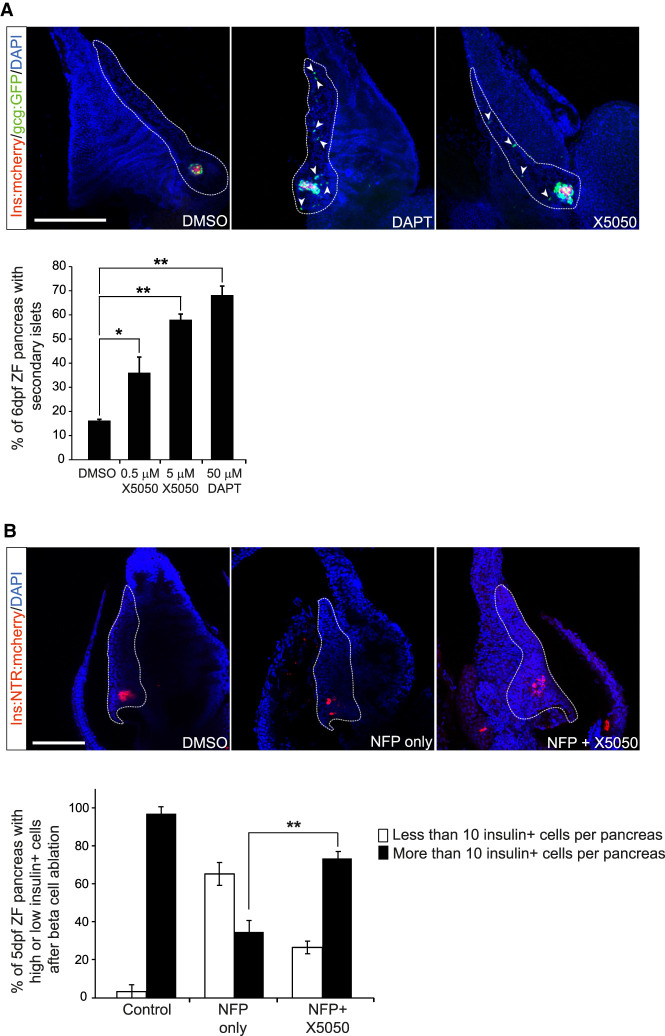
REST regulation of endocrine differentiation is conserved in zebrafish. (*A*) Embryos of *Ins:mCherry/Gcga:GFP* double-transgenic zebrafish line treated with 50 µM DAPT (Notch inhibitor used as positive control), 0.5 or 5 µM X5050, or vehicle (DMSO, negative control) from 3 dpf until 6 dpf. After drug treatment at 6 dpf, zebrafish pancreas was dissected, and the presence or absence of secondary islets was quantified. Arrows show representative secondary islets of double-transgenic zebrafish embryos (*Ins:mCherry/Gcga:GFP*; [blue] DAPI). The graph shows the percentage of zebrafish with detectable secondary islets in each condition. *n* = 20–25 fish per each condition. (*B*) An *Ins:NTR-mCherry* line was used to selectively ablate β cells upon treatment with 5 µM nifurpirinol (NFP) (Pisharath et al. 2007; Bergemann et al. 2018) in 3-dpf embryos; 24 h later after complete β-cell ablation of the principal islet, embryos were exposed to 5 µM ×5050 or vehicle, and β cells were analyzed 36 h later. Representative images of β-cell regeneration in *Ins:NTR-mCherry* embryos treated with vehicle (DMSO), with NFP only, or with NFP and X5050. (Red) Insulin, (blue) DAPI. The graph shows the percentage of pancreas showing >10 insulin-expressing cells in every condition. *n* = 32–36 fish per each condition. Scale bars, 200 µm. Error bars are SEM. (**) *P* < 0.01, (*) *P* < 0.05, χ^2^ test.

To study REST function in zebrafish, we used a double transgenic line in which glucagon- and insulin-expressing cells show green and red fluorescence, respectively (*Ins:mcherry/Gcga:gfp*). We treated zebrafish embryos at 3 dpf with X5050 for 3 d or with the Notch inhibitor DAPT as a positive control (Parsons et al. 2009), and at 6 dpf, we dissected the pancreas to quantify secondary islet formation as a readout for endocrine cell differentiation. Secondary islets are normally apparent in ∼10% of control larvae at 5 dpf, and this percentage increases gradually thereafter (Parsons et al. 2009). Compared with DMSO controls, X5050-treated 6-dpf embryos displayed a dose-dependent increase in secondary islet formation (5 µM and 50 µM: 2.2-fold ± 0.4-fold and 3.5-fold ± 0.1-fold increase; SD, Student's *t*-test *P* < 0.05 and *P* < 0.01, respectively), which was comparable with 50 µM DAPT (4.2-fold ± 0.4-fold, *P* < 0.01) (Fig. 5A). These results suggest that REST regulation of pancreatic endocrine differentiation is conserved in zebrafish and that this process can be manipulated through chemical inhibition.

We next investigated whether REST inhibition could accelerate β-cell neogenesis after β ablation with nifurpirinol (NFP), using *Ins:NTR-mcherry* transgenic zebrafish (Bergemann et al. 2018). In this model, 3-dpf embryos are treated with NFP, causing ablation of >95% of β cells in 24 h and full recovery of β-cell mass in 48–72 h (Bergemann et al. 2018). We note that at 3 dpf, all β cells form part of principal islets. After washing NFP, we ascertained complete β-cell ablation with a stereomicroscope and then treated embryos with X5050 or vehicle, and 36 h later, we examined which embryos had recovered >10 β cells, a threshold that enables unequivocal distinction from complete ablation. We observed that 73.4% ± 3.3% of X5050-treated embryos showed >10 insulin-positive cells in the principal islet, whereas this was only seen in 34.7% ± 6.1% of controls (mean and SD of 34–38 embryos in each group, χ^2^ test, *P* < 0.01) (Fig. 5B). Thus, REST inhibition promoted β-cell formation in a zebrafish embryo regeneration model.

### REST inhibition in human organoids

**Figure 6.**
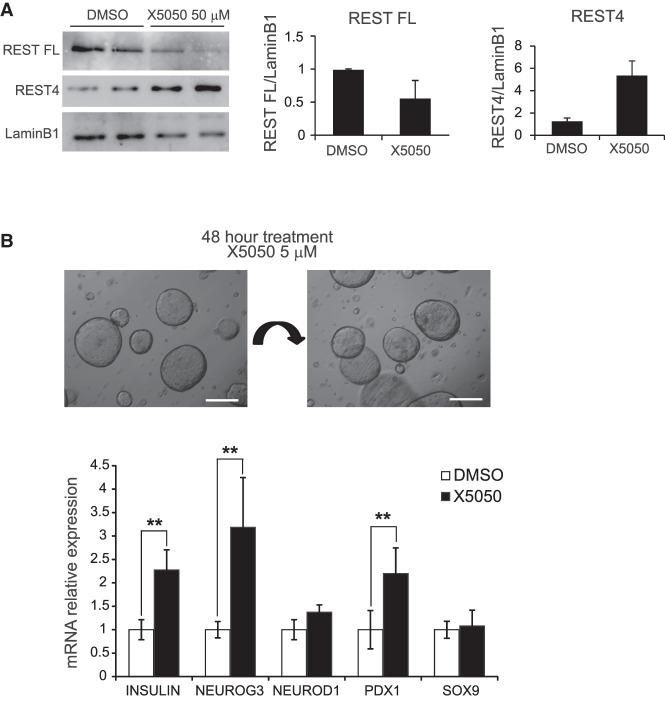
REST chemical inhibition in human pancreatic organoids. (*A*) Western blot analysis of REST FL (full-length) and REST4 protein levels in PANC1 cells treated with X5050 50 µM or DMSO (control). (Lamin B1) Loading control. Bar plot shows the quantification of the Western blot for REST. (*B*) Human organoids generated from pancreatic exocrine fractions from two cadaveric donors were treated at passage 3 for 48 h with 5 µM X5050 or DMSO (control vehicle). qPCR analysis of mRNA for indicated genes, relative to TBP. Scale bars, 200 µm. Error bars are SD. Student's *t*-test, (**) *P* < 0.01.

We next explored the impact of REST manipulation in human cells. We first validated that 24-h treatment of an immortalized duct cell line (PANC-1) with X5050 caused an ∼50% reduction of REST full-length protein, as well as a relative increase in the REST4 isoform, as previously described (Fig. 6A; Charbord et al. 2013). Next, we studied ex vivo organoid cultures from human adult ducts isolated from the exocrine fraction of the pancreas of cadaveric donors. Organoids were generated and expanded as previously described (Boj et al. 2015), and experiments were performed at passages 3–4 (Fig. 6B). Currently, the efficiency of endocrine differentiation from published ex vivo pancreas organoid protocols is still limited (Huch et al. 2013; Boj et al. 2015; Loomans et al. 2018). We thus investigated if REST inhibition in pancreatic human organoids could promote the activation of pancreatic endocrine lineage genes. We treated human organoids with X5050 for 48 h and observed only rare endocrine cells in treated and nontreated organoids. However, X5050-treated organoids showed induction of *INS*, *NEUROG3*, and *PDX1* mRNA levels (2.27-fold ± 0.43-fold, 3.18-fold ± 1.06-fold, and 2.18-fold ± 0.54-fold increased vs. DMSO, respectively; SD, Student's *t*-test, *P* < 0.01), whereas the duct cell marker *SOX9* mRNA did not change (Fig. 6B). These results therefore show that chemical interference of REST in adult human pancreas organoids did not lead to β-cell formation, consistent with genetic findings in adult mice, although it induced the transcription of endocrine genes.

## Discussion

Despite early suggestions that REST could be important for pancreatic endocrine differentiation during embryonic development (Martin et al. 2008, 2012; van Arensbergen et al. 2010), conditional ablation of *Rest* in the mouse pancreas unexpectedly showed modest gene expression differences and no quantitative changes in endocrine cell formation (Martin et al. 2015). This result clearly did not suggest a major regulatory role in pancreas endocrinogenesis. We have now combined genetic and chemical perturbations to show that REST plays a key evolutionary conserved role to modulate the generation of endocrine cells during pancreas organogenesis. We define for the first time a blueprint of direct REST target genes in the embryonic pancreas that underpin this regulatory function. We further show that the capacity to increase endocrinogenesis upon REST inactivation in duct cells decreases during postnatal life, whereas REST inhibition in human adult pancreas organoids influenced expression of endocrine genes but did not trigger endocrine cell formation. These findings therefore establish REST as an important regulator of endocrinogenesis during embryonic pancreas development.

During pancreas development, a subset of HNF1B+ duct–endocrine bipotent progenitors that form a tubular plexus trigger an endocrine gene program, whereas others give rise to mature ductal cells (Solar et al. 2009; Bankaitis et al. 2015). The mechanisms that underlie this binary lineage choice in a seemingly uniform pool of progenitors is unclear. Our results suggest that REST restrains the frequency with which bipotent progenitors consolidate an endocrine vs. duct fate. On the other hand, the fact that REST deficiency did not cause an en masse conversion of bipotent progenitors into endocrine cells is consistent with the notion that REST is not the sole guardian of endocrine differentiation but instead acts in concert with other positive and negative regulators to define a differentiation probability.

Genetic experiments have indeed revealed numerous transcriptional regulators that promote pancreatic endocrine cell formation, including NKX6-1, NKX2-2, NEUROG3, HNF1B, and INMS1, among others (Sussel et al. 1998; Gradwohl et al. 2000; Osipovich et al. 2014; De Vas et al. 2015). Among DNA binding factors that suppress endocrinogenesis, Hippo-responsive TEAD-YAP complexes are an integral component of pancreatic multipotent progenitor enhancers and plausibly counteract endocrine differentiation by promoting a progenitor transcriptional state (Cebola et al. 2015; Mamidi et al. 2018; Rosado-Olivieri et al. 2019). Notch-responsive transcriptional repressors, notably HES1, bind near endocrine genes, where they are likely to exert direct transcriptional repression (Jensen et al. 2000; de Lichtenberg et al. 2018). An important unsolved question is how REST interplays with such inhibitory and positive regulators at different stages to ensure a timely and balanced generation of endocrine cells.

The inhibitory function of REST has potential implications for efforts to enhance endocrinogenesis in various in vivo or in vitro settings. Current pancreas organoid models are limited because existing protocols largely recapitulate exocrine cell expansion. On the other hand, our experiments showed that REST derepression enhanced endocrinogenesis in embryonic progenitors and even early postnatal pancreas but was clearly less efficient in the adult differentiated pancreas. This may mean that REST restrains endocrine differentiation in progenitors that express positive endocrine regulators and have the appropriate epigenetic competence, whereas mature duct epithelial cells may lack these properties. Nonetheless, the observation that REST inhibition did elicit increased expression of islet endocrine genes in human exocrine organoids, together with the recent observation that it can enhance transcription factor-mediated reprogramming of mouse adult exocrine cells (Elhanani et al. 2020), suggests that REST modulators may form part of an arsenal for future manipulations to promote endocrinogenesis in experimental model systems or replacement therapies.

## Materials and methods

### Mouse models

All experiments were approved by the Institutional Animal Care Committee of the University of Barcelona. Mice with *Rest* exon 4 floxed allele (*Rest*^*LSL*^) (Yamada et al. 2010) were crossed to *Pdx1*-Cre (Gu et al. 2002) or *Hnf1b*-CreERT2 (Solar et al. 2009) and Rosa26-LSL-RFP transgenic lines (Luche et al. 2007). We also generated *Rest*^*LSL*^ mice carrying a different *Pdx1*-Cre transgene (Hingorani et al. 2003) and confirmed increased endocrine cell mass in adult mice as well as increased NEUROG3+ cells at E18.5. To induce recombination in triple transgenics (*Hnf1b-CreERT2;Rosa26RFP;*Rest^LSL^ or *Hnf1b*-CreERT2;Rosa26^RFP^ control mice), 20 mg of tamoxifen (Sigma T5648) was administered by gavage to the mother at days 1 and 3 after delivery. Mice were then sacrificed at 30 d of age. For adults, tamoxifen was given by gavage in three doses (20 mg, 20 mg, and 10 mg) over 1 wk to 8- to 12-wk-old mice, and mice were analyzed 4 wk later. Oligonucleotides used for genotyping are in Supplemental Table S8.

### Dissociation and FACS analysis of pancreatic cells

Adult and E18.5 mouse pancreas from *Sox9*-eGFP transgenics (Gong et al. 2003) were digested in 1.4 mg/mL collagenase-P (Roche) for 20–30 min at 37°C. Peripheral acinar-ductal units, depleted of endocrine islets, were prepared as previously described (Wang et al. 2013). Tissue was filtered through 600-μm and 100-μm polypropylene meshes (BD), and peripheral acinar-ductal units were further dissociated in diluted TrypLE (Invitrogen) for 5 min at 37°C. Dispersed cells were filtered through a 40-μm polypropylene mesh (BD) before FACS sorting.

### Immunoblots

Nuclear extracts were prepared as previously described (Maestro et al. 2003) and separated on a 7% SDS-PAGE gel and transferred to an Immobilon polyvinylidene difluoride membrane (Millipore). Immunodetection was performed with mouse 12C11 anti-REST (1:1000) or rabbit anti-LaminB1 (1:2000; Cell Signaling). Quantification was performed with ImageJ-Fiji.

### RNA analysis

RNA was isolated using the RNeasy minikit (Qiagen) or TRIzol followed by DNase I treatment (Invitrogen). RNA was reverse-transcribed with SuperScript III reverse transcriptase (Roche) and random hexamers, and qPCR was performed on a 7900 real-time PCR system (Applied Biosystems) using Power SYBR green (Applied Biosystems). Oligonucleotides are shown in Supplemental Table S8.

### RNA-seq

DNase-treated RNA (RIN > 8) was generated from three E18.5 pancreas for each genotype and used for 100-bp paired-end read Illumina sequencing. Reads were aligned to the NCBI36/mm9 genome using STAR (v2.3.0) (Dobin et al. 2013) with default parameters, allowing only uniquely mapped reads. The resulting bam files were used to quantify gene expression using FeatureCounts (v1.5) using UCSC mm9 reference gene annotations. Differential expression analysis was performed using DESeq2 (Love et al. 2014) using an adjusted *P*-value < 0.05 cutoff.

### ChIP-seq

ChIPs were from dissected E13.5 pancreatic buds and were performed as described by van Arensbergen et al. (2010, 2013).

ChIP DNA (1–2 ng) from two independent pools of E13.5 pancreas were used for ChIP sequencing of single-end 50-bp reads. Reads were aligned to NCBI36/mm9 genome using Bowtie2 (v2.2.5) allowing for one mismatch. Bam files were filtered to retain reads with a MAPQ ≥ 10. Bam files from biological replicates were pooled using samtools, and peaks were called using MACS2 (v2.1.0) using default parameters. Input DNA was used to define significant peaks at a false detection rate of <0.05.

### Functional gene annotations

GSEA was performed in preranked lists and analyzed with 1000 permutations. Differentially expressed genes were functionally annotated using Gorilla (Eden et al. 2009), and REVIGO (Supek et al. 2011) was used to visualize most significant terms in each GO cluster.

### ATAC-seq profiles around transcription factor binding sites

To plot ATAC-seq profiles around transcription factor binding sites (TFBSs), we conducted footprinting with HINT from the RGT library (v0.13.1) (Li et al. 2019). An ATAC-seq model and paired-end data were specified for tool execution, using aligned read BAM files and ATAC-seq peaks in BED format. Once footprints were called, overlapping TFBSs were found using the RGT-matching motif tool and the complete JASPAR motifs database. For REST (MA0138.2), we selected only footprints overlapping a REST ChIP-seq peak using ChIPpeakAnno (v3.18.1), GenomicRanges (v1.36.0), and Bioconductor (v3.9.0) R (v3.6.3) and lifting to mm10. Normalized ATAC-seq profiles around TFBSs of interest were generated with HINT for a 500-bp window size, enabling ATAC-seq bias correction.

### REST binding enrichment in differentially expressed genes

Genomic regions were associated with genes using GREAT v3.0, applying default parameters to the basal plus extension association rule (McLean et al. 2010). Proximal and distal REST-bound regions were defined as <5 kb or >5 kb from the transcriptional start sites, respectively. REST-bound genes were annotated based on H3K27me3 enrichment (van Arensbergen et al. 2010).

### Motif analysis

De novo and known motifs of REST-bound regions were analyzed with HOMER, using a 500-bp window centered on the REST peak.

### Immunolocalization methods

Paraffin-embedded pancreas were processed for immunolocalization as described by Maestro et al. (2003). Whole-mount staining of E18.5 pancreas was performed as previously described (Ahnfelt-Rønne et al. 2007) without TSA amplification. For β-cell mass measurements in 3-mo-old mice, 4-µm sections were obtained at 150-µm intervals, and 21–24 sections per pancreas were analyzed by immunofluorescence for insulin and DAPI. Images were taken by automated capturing and reconstruction of 10 × 10 frames using a Leica SP confocal microscope. Insulin-positive and total tissue areas (measured by DAPI saturation) were determined by ImageJ. Islet size distribution was quantified with an automated ImageJ plugin. Antibodies are shown in Supplemental Table S9.

### Human organoid culture

Human exocrine tissue, obtained from the discarded fraction after human islet purifications from cadaveric organ donors (Gmyr et al. 2000), was used only if islets were insufficient for clinical transplantation and if scientific research was granted according to national French regulations. Ethical approval for processing pancreatic samples from deidentified organ donors was granted by the Clinical Research Ethics Committee of Hospital Clinic de Barcelona (HCB/2014/0926 and HCB/2014/1151).

Tissues were minced and digested with 5 mg/mL collagenase II (Gibco) in human complete medium for 30 min to 1 h at 37°C. The material was further digested with TrypLE (Gibco) for 5 min at 37°C, embedded in GFR Matrigel (Kerr Conte et al. 1996), and cultured in human organoid expansion medium (Boj et al. 2015). After three passages, we treated organoids with X5050 (Calbiochem) in human complete media for 48 h before RNA analysis.

### Zebrafish studies

*Tg(gcga:GFP), Tg(T2KIns:hmgb1-mcherry)*, and *Tg(ins:NTR-mCherry)* were obtained from Isabelle Mandfroid (University of Liege, Belgium) (Parsons et al. 2009; Bergemann et al. 2018). Double-transgenic *Tg(gcga:GFP)/Tg(T2KIns:hmgb1-mCherry)* embryos were incubated from 3 dpf to 6 dpf at 28°C in the dark in 50 µM DAPT (Tocris) and 0.5 and 5 µM X5050 (Calbiochem) (Charbord et al. 2013) or 1% DMSO in E3 (Parsons et al. 2009). Zebrafish were then fixed overnight in 4% paraformaldehyde; pancreas was dissected for confocal image analysis.

*Tg(Ins:NTR-mCherry)* was used for β-cell ablation studies upon treatment with 5 µM nifurpirinol (NFP) (Bergemann et al. 2018) in 3-dpf embryos. In this model, 24 h after NFP treatment, >95% of the β cells are ablated and β-cell mass recovered in 48–72 h (Parsons et al. 2009; Bergemann et al. 2018). NFP-treated embryos were washed and treated with 5 µM X5050 or DMSO. β-Cell regeneration was analyzed after 36 h. After drug treatment, 5-dpf zebrafish were fixed overnight in 4% paraformaldehyde, and pancreas was dissected for confocal image analysis.

To microdissect pancreas, fixed embryos were placed in PBS on an agarose-lined plate. Then, using pulled capillaries as tools, first the yolk and then the whole foregut were pried away from the embryo. The pancreas was placed islet-down on a coverslip and dried by removing all excess PBS. This coverslip was then mounted onto a microscope slide. Water was introduced under the cover slip to rehydrate the sample.

Confocal Z-series stacks were acquired on a Leica SP5 confocal microscope. Maximum projections were obtained by LAS AF software. To count endocrine cells, we used a double-transgenic line. *Tg(gcga:GFP)* and *Tg(T2KIns:hmgb1-mCherry)*, where glucagon-positive cells were green and insulin-positive cells were red. Upon maximum projections of Z-series of the entire pancreas, the presence or absence of secondary islets was computed.

### Statistics

Statistical analyses were performed using either R or GraphPad Prism 6. Statistical significance was calculated with a Fisher exact test or an unpaired, two-tailed Student's *t*-test with data expressed as mean ± SEM unless otherwise specified. *P*-values < 0.05 were considered significant.

### Data availability

ChIP-seq and RNA-seq data sets are available in GSE179120.

## Supplementary Material

Supplemental Material
