# Supplementary material for: REST is a major negative regulator of endocrine differentiation during pancreas organogenesis
Source: Genes Dev. 2021 Sep 1;35(17-18):1229–42. doi: 10.1101/gad.348501.121 (PMC8415321; doi:10.1101/gad.348501.121)
Supplement: Supplemental Material [file supp_35_17-18_1229__DC1.html]

REST is a major negative regulator of endocrine differentiation during pancreas organogenesis — Supplemental Material 

# REST is a major negative regulator of endocrine differentiation during pancreas organogenesis

## Supplemental Material

- supplemental\_Figures.pdf
- Supplemental\_table1.xlsx
- Supplemental\_table2.xlsx
- Supplemental\_table3.xlsx
- Supplemental\_table4.xlsx
- Supplemental\_table5.xlsx
- Supplemental\_table6.xlsx
- Supplemental\_table7.xlsx
- Supplemental\_table8.xlsx
- Supplemental\_table9.xlsx
