# Supplementary material for: REST is a major negative regulator of endocrine differentiation during pancreas organogenesis
Source: Genes Dev. 2021 Sep 1;35(17-18):1229–42. doi: 10.1101/gad.348501.121 (PMC8415321; doi:10.1101/gad.348501.121)
Supplement: Supplemental Material [file supp_gad.348501.121_supplemental_Figures.pdf]

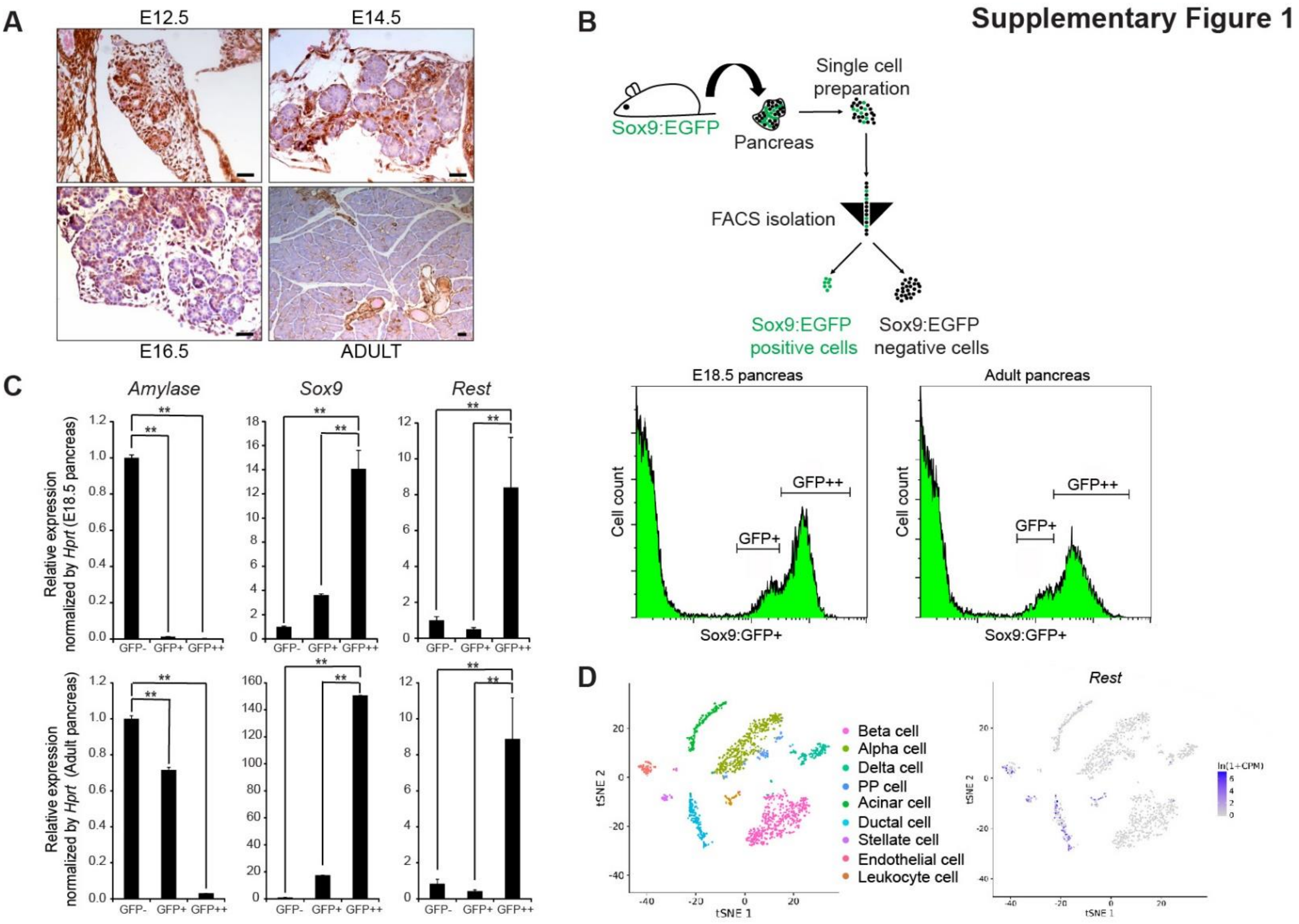

Supplementary Figure 1. REST expression in the pancreas. (A) IHQ staining to detect REST protein levels during pancreas development (E12.6, E14.5 and E16.5) and adult. Scale bars= 100 µm. (B) Schematic of the sorting strategy to isolate ductal cells from E18.5 and adult pancreas from *Sox9*-GFP transgenic mice (Gong et al. 2003) . Representative sorting plots and gating strategy to isolate high (GFP++), low (GFP+) and negative (GFP-) Sox9-GFP expressing cells. (C) qPCR analysis of *Rest* expression levels in FACS isolated ductal cells at E18.5 and adult pancreas, show increased *Rest* mRNA in duct-enriched (*Sox9* expressing cells) vs non-duct fractions (mainly acinar cells, *Amylase* expressing cells). (D) Analysis of scRNA-seq datasets (Tabula Muris et al. 2018), show *Re*st expression in adult pancreatic ductal cells and non-epithelial cells, but not in insulin- and glucagon-expressing cells. Error bars are SEM. ** p ≤ 0.01.

Supplementary Figure 2

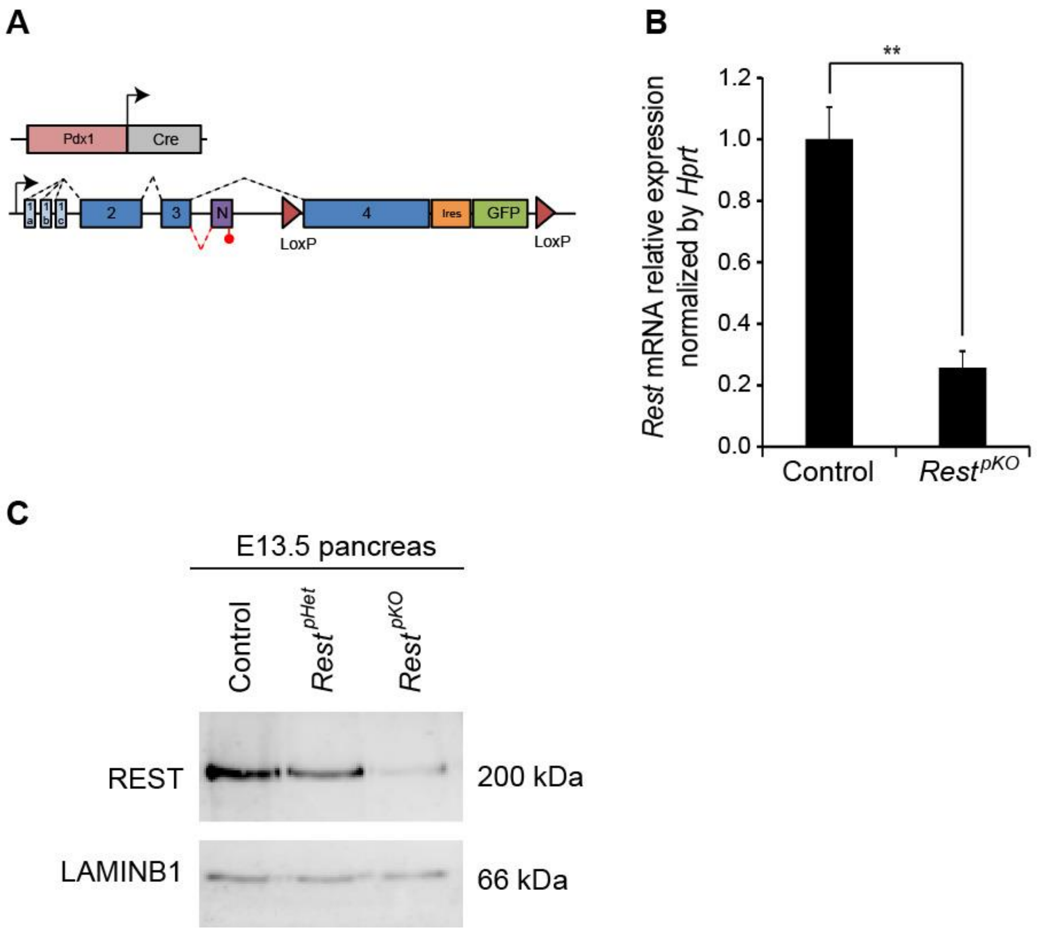

Supplementary Figure 2. *Rest* pancreatic KO mouse model (*Rest*^{pKO}). (A) Schematic of the two genetic models to delete *Rest* from pancreatic epithelial cells. (B) qPCR analysis of *Rest* mRNA levels in E13.5 pancreas upon deletion. Results are normalized by *Hprt*, n = 3 independent embryos in each group. Error bars are SEM. ** p≤ 0.01. Note that pancreas contains mesenchymal cells at this stage where excision is not expected. (C) Western blot images of REST protein levels in the pancreas of control, *Rest*^{pHet}, carrying heterozygous LoxP allele and Pdx1-Cre, and *Rest*^{pKO}. LaminB1 is a loading control.

Supplementary Figure 3

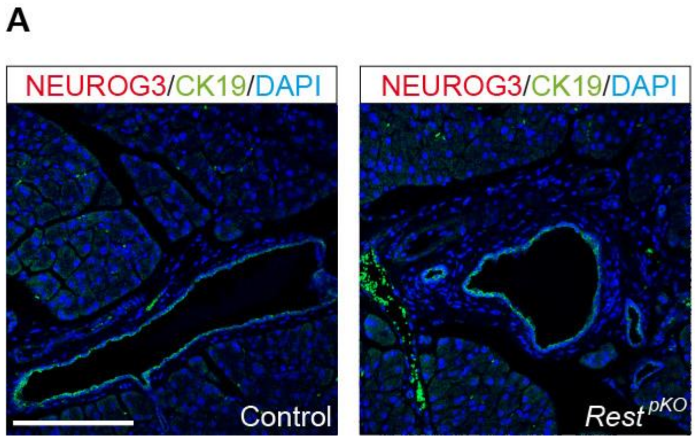

Supplementary Figure 3. NEUROG3 expression in adult *Rest*^{pKO} mice. Representative immunofluorescence staining for *Neurog3* (red), CK19 (green) and DAPI (blue) in adult pancreas from control *and Rest*^{pKO} mice shows absence of NEUROG3 positive cells. These findings indicate that REST-independent inhibitory mechanisms, and or lack of *Neurog3* activating mechanisms, acquire prominence during postnatal life. Scale bars = 200 µm.

A

Supplementary Figure 4

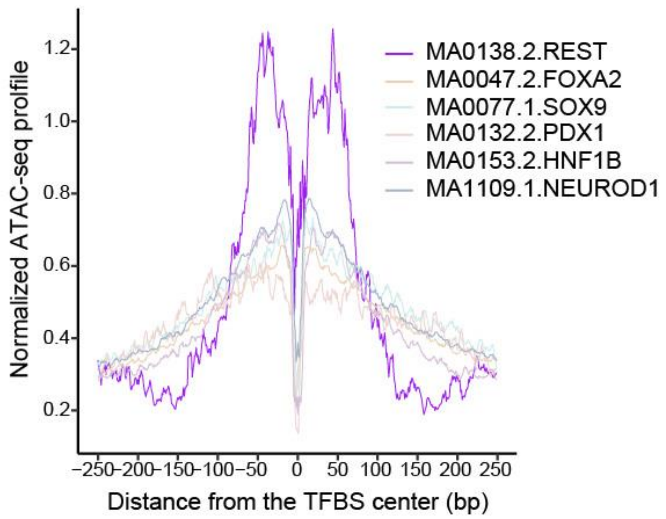

Supplementary Figure 4. Integration of REST-bound regions with ATAC-seq profiles from E13.5 pancreas shows a distinct, more narrow chromatin accessibility footprint of REST-bound regions relative to activating transcription factors.

Supplementary Figure 5

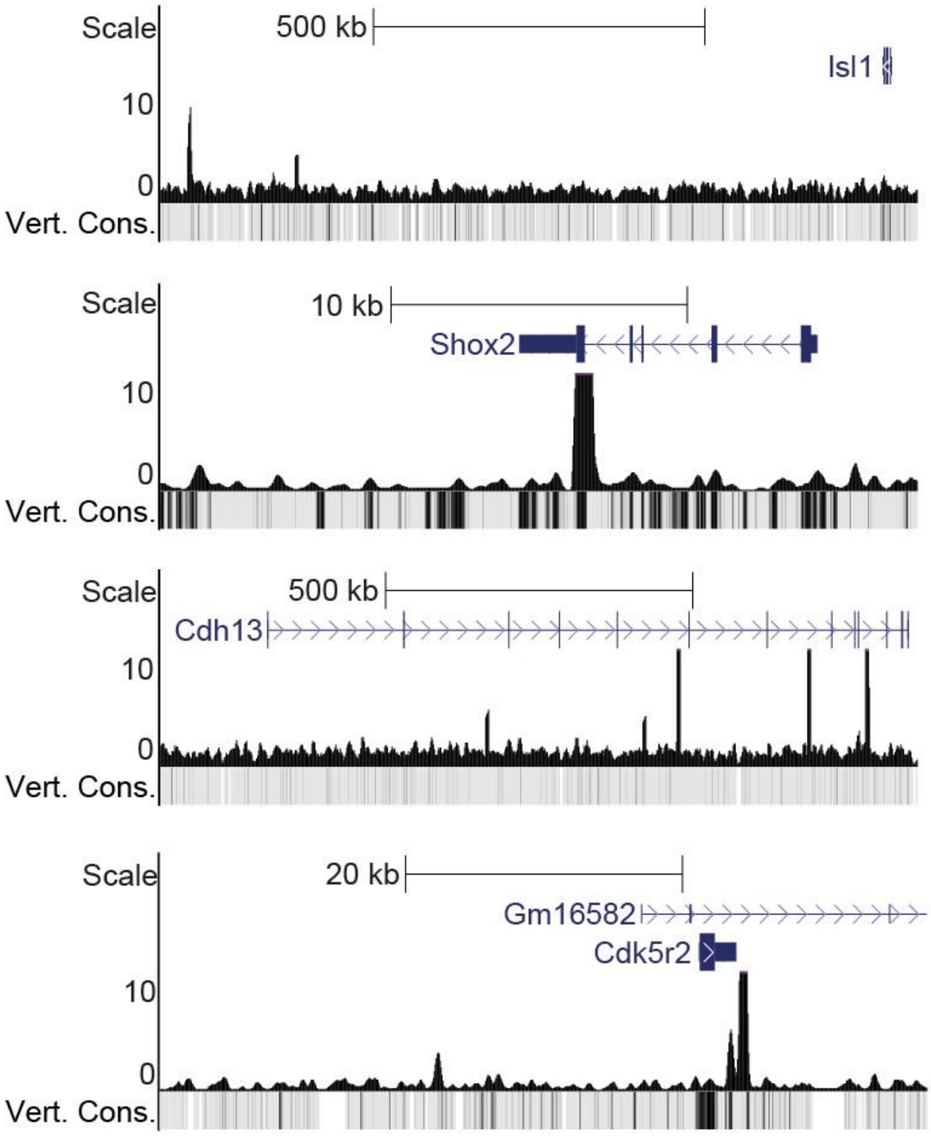

Supplementary Figure 5. Representative tracks of regions bound by REST in embryonic pancreas but not in mESC and mNSC datasets examined. Note that the distal binding site in the *ISL1* locus is located in a broad gene-poor region that contains regulatory elements associated with *ISL1*. The Y axes show -Log10 P values.

Supplementary Figure 6

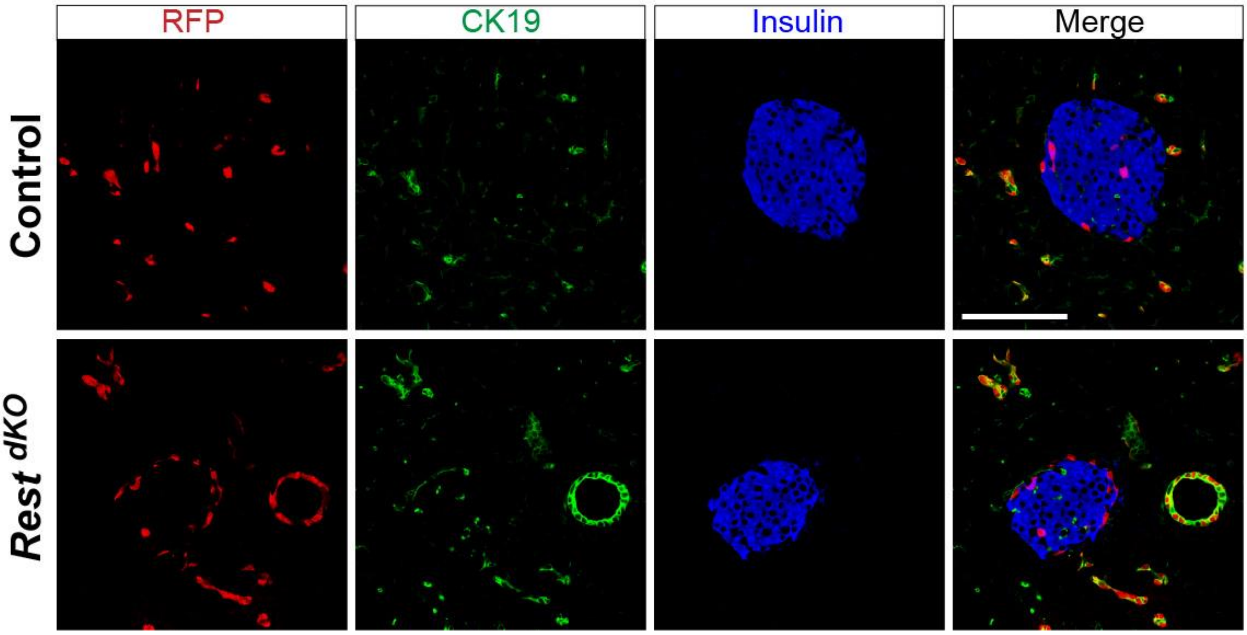

Supplementary Figure 6. *Rest* pancreatic ductal KO in adult mice (*Rest*^{dKO}). Representative immunofluorescence staining for *Insulin* (blue), CK19 (green) and RFP (red) in adult pancreas of 12-week-old mice from control (*Hnf1b*-CreERT2;Rosa26^{RFP}) *and Rest*^{dKO} (*Hnf1b*-CreERT2;*Rest LSL*;Rosa26^{RFP}) genotypes. CK19+ cells in large and small ducts showed >60% RFP+ labelling in both genotypes, but no differences in Insulin+/RFP+ cells between control *and Rest*^{dKO} mice were observed, thus ruling out that *Rest*-deficiency at this stage leads to frequent duct-to-endocrine cellular transitions. Scale bars = 100 µm.
